# Psychological stress affecting the size and resolution of an intermittent post-traumatic vascular lesion of the temporalis muscle

**DOI:** 10.1590/1677-5449.202501052

**Published:** 2026-04-27

**Authors:** Harshit Arora, Vasudha Sharma, Lavanya Arora, Waryaam Singh, Rajesh Pasricha

**Affiliations:** 1 Dayanand Medical College & Hospital, Ludhiana, Punjab, India.; 2 University of Kentucky, Department of Neurosurgery, Lexington, KY, USA.; 3 Teerthanker Mahaveer Medical College & Research College, Moradabad, Uttar Pradesh, India.; 4 Mayo Clinic, Department of Infectious Diseases, Rochester, MN, USA.; 5 Post Graduate Institute of Medical Education & Research, Chandigarh, India.; 6 Satyam Hospital and Trauma Center, Jalandhar, Punjab, India.

**Keywords:** vascular malformation, psychological stress, trauma, temporalis muscle, size variation, malformação vascular, estresse psicológico, trauma, músculo temporal, variação de tamanho

## Abstract

Vascular anomalies are disorders of arteries or veins which are further sub-classified as vascular malformations or vascular tumors. Vascular malformations are attributed to disorderly vascular morphogenesis, trauma, or hormonal imbalances. We present a rare case of a patient in their 30s who presented to the outpatient department with a localized post-traumatic soft tissue swelling in the temporalis region, which was soft and non-pulsatile with occurrences in periods of acute emotional stress, followed by spontaneous resolution when the same was negated by the patient. The diagnosis was established on the basis of magnetic resonance imaging and contrast enhanced computed tomography. We add to the existing limited literature on intramuscular vascular malformations of the temporalis muscle and highlight the effect of emotional stress and anxiety on vascular anomalies.

## Resumo

As anomalias vasculares referem-se a distúrbios de artérias ou veias, podendo ser subclassificadas como malformações vasculares ou tumores vasculares. As possíveis causas de malformações vasculares incluem morfogênese vascular desordenada, trauma ou desequilíbrios hormonais. Apresentamos o caso raro de um paciente na faixa etária dos 30 anos que se apresentou ao ambulatório com edema localizado de tecido mole pós-traumático na região temporal, de consistência macia e não pulsátil, com ocorrência em períodos de estresse emocional agudo e resolução espontânea quando esse fator era negado pelo paciente. O diagnóstico foi estabelecido com base em ressonância magnética e tomografia computadorizada com contraste. Este relato contribui para a limitada literatura existente sobre malformações vasculares intramusculares do músculo temporal e destaca o efeito do estresse emocional e da ansiedade nas anomalias vasculares.

## INTRODUCTION

Vascular anomalies are disorders of the vascular system, arteries, veins, and lymphatics, which can be subclassified into vascular malformations (VM) and vascular tumors. While the former are caused by inadequate vascular morphogenesis, tumors like hemangiomas are attributed to cellular hyperplasia.^1^ Intramuscular vascular lesions are rare, representing <1% of all VM, with approximately 14% occurring in the head and neck region. These typically involve the face, buccal mucosa, lips, palate, and gingiva, as well as the pharynx and larynx, and only rarely arise intramuscularly, most often in the masseter muscle.^2^ Trauma and hormonal imbalances are the most common contributing factors in VM.^3^ VM in the temporal region usually present as soft localized swellings and can be associated with pain and tenderness. These lesions are diagnosed using magnetic resonance imaging (MRI) or contrast enhanced computed tomography (CECT), with confirmatory diagnosis established on histopathological evaluation.^4^ Treatment involves either surgical resection, embolization, or a combination of both modalities.^5^

While psychological stress has been shown to be linked with inflammation,^6-8^ our case represents a unique presentation of VM, with size variation and spontaneous resolution linked to emotional stress.^9^ We present the case of a patient in their 30s with an intermittent swelling in the left temporal region, associated with periods of stress and nervousness, followed by spontaneous resolution of the lesion over the passage of a few hours.

## CASE REPORT

**Figure 1:**
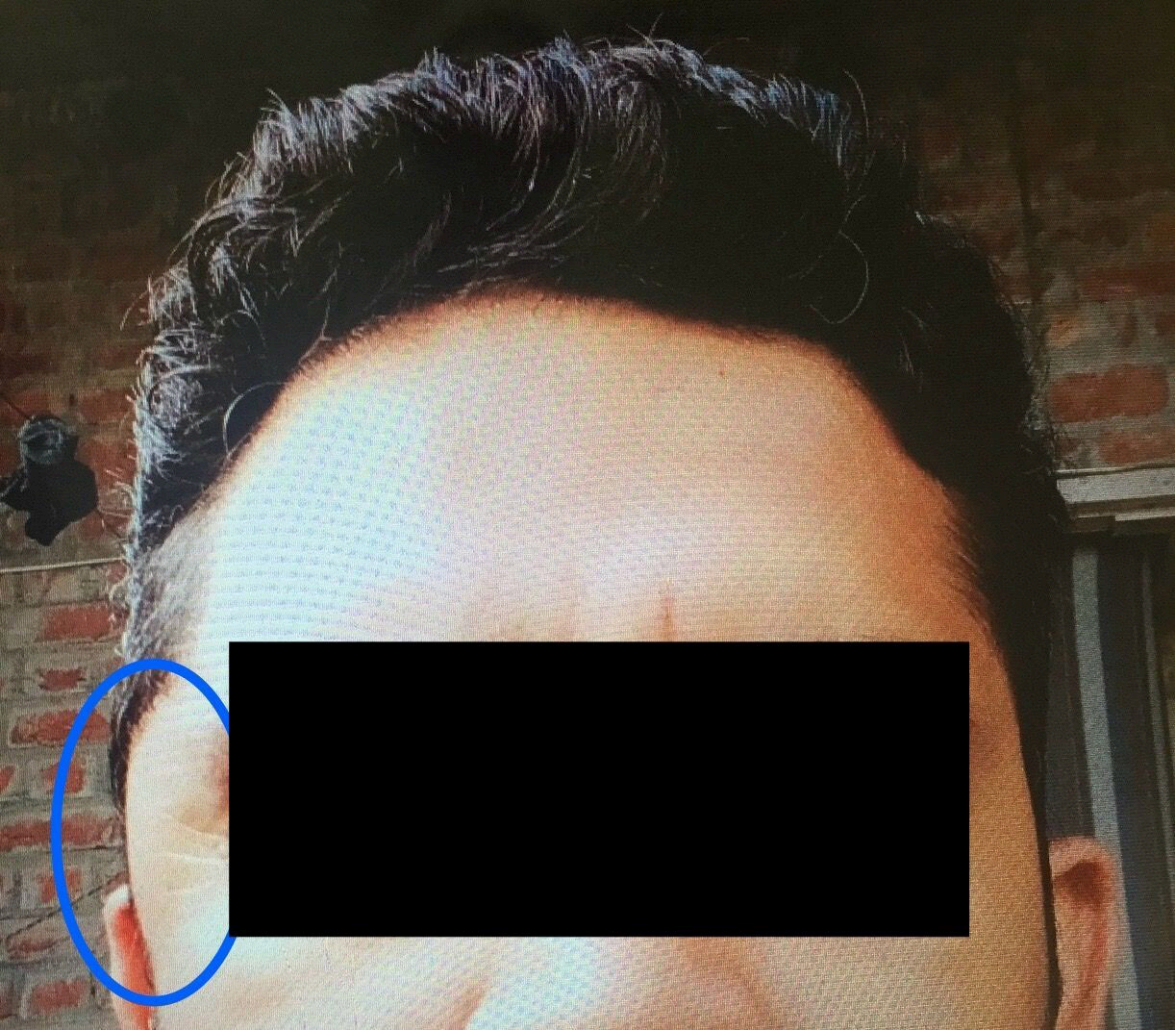
A 7 cm x 5.5 cm smooth, soft to firm in consistency, non-pulsatile, immobile swelling in the left temporal region (circled in blue).

A patient in their 30s presented to a tertiary care hospital in Northern India with a chief complaint of intermittent swelling on the left side of the head for the past 4-5 months, as shown in Figure 1. The swelling appeared sporadically with a frequency of once or twice per fortnight and spontaneously resolved within a few hours of occurrence. The patient further reported that these episodes were associated with periods of acute emotional stress and were correlated with higher levels of blood pressure. There was no diurnal or seasonal variation. The swelling was associated with dull aching pain and heaviness in the region and difficulty opening the mouth and left eye. Informed consent was obtained from the patient. The study was exempted from the need for ethics approval by the institutional ethics committee because it is a case report. The Helsinki Declaration and local ethical guidelines were followed.

The patient reported a similar episode 4 years prior to the index presentation which had resolved spontaneously without any medical intervention. There was no history of vertigo, seizures, or loss of consciousness, or any other significant medical history. The patient denied use of any long-term medications. There had been three incidents of localized trauma to the left side of the head occurring 25 years, 20 years, and 12 years prior to presentation. The second incident (20 years ago) was associated with loss of consciousness, while the others were insignificant. No bleeding or external wounds were reported across these three incidents. The patient was a non-smoking teetotaler and denied any illicit drug use.

**Figure 2:**
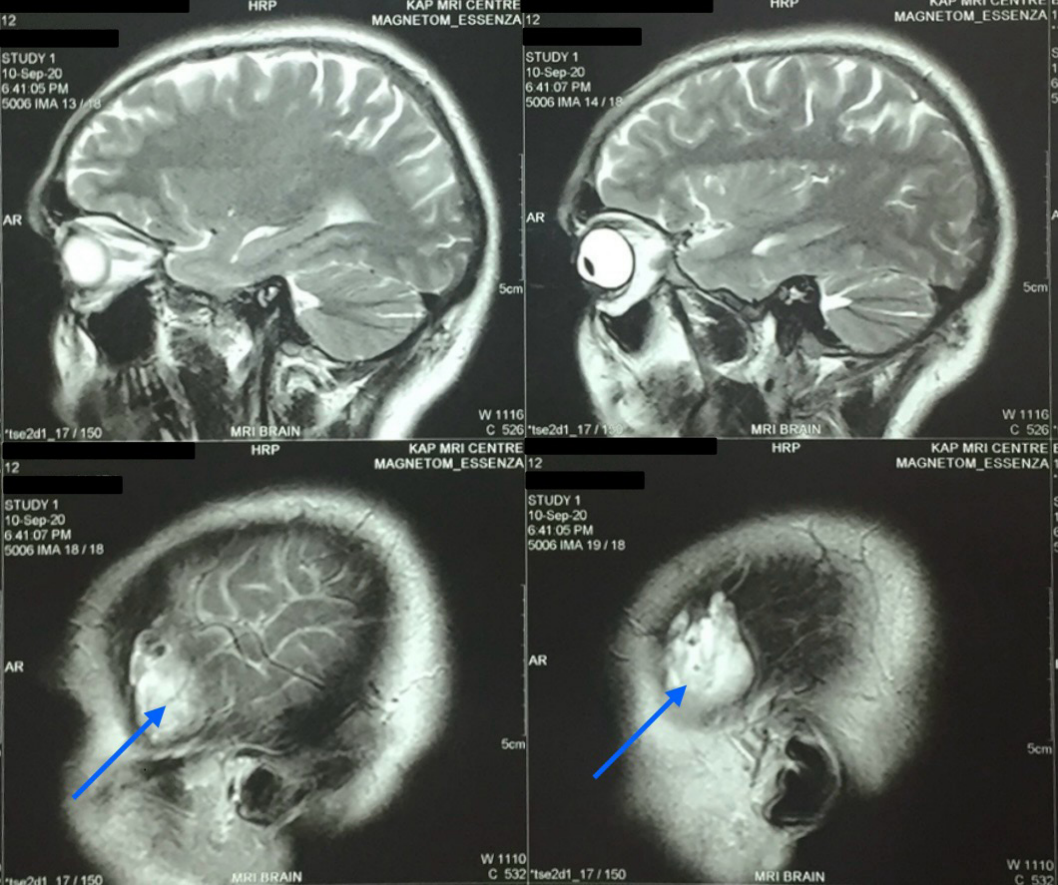
A 4.3 x 3.3 x 1.3 cm heterogenous lesion in the temporalis muscle, hyperintense on T2 weighted brain and neck MRI (sagittal view, indicated with blue arrows).

**Figure 3:**
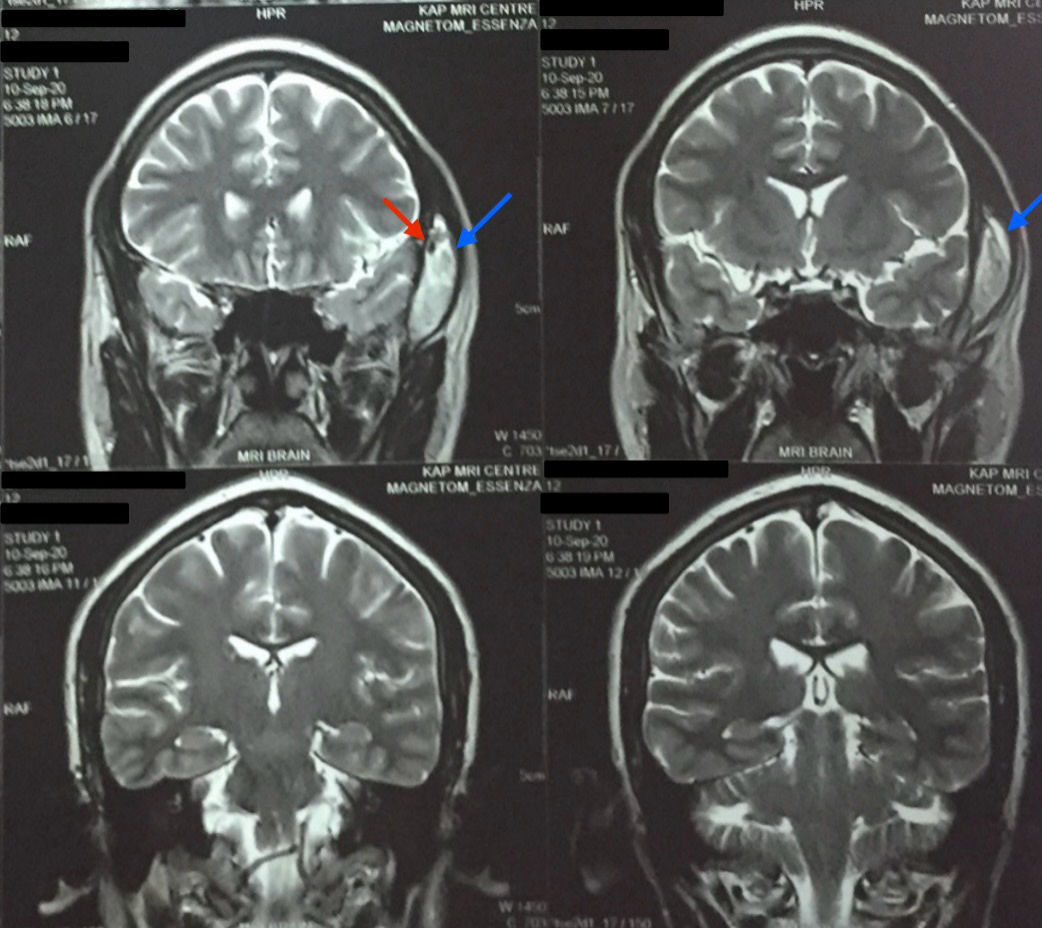
A 4.3 x 3.3 x 1.3 cm heterogenous lesion in the temporalis muscle (indicated with blue arrows), hyperintense on T2 weighted brain and neck MRI (coronal view) with foci of calcification (indicated with red arrow).

**Figure 4:**
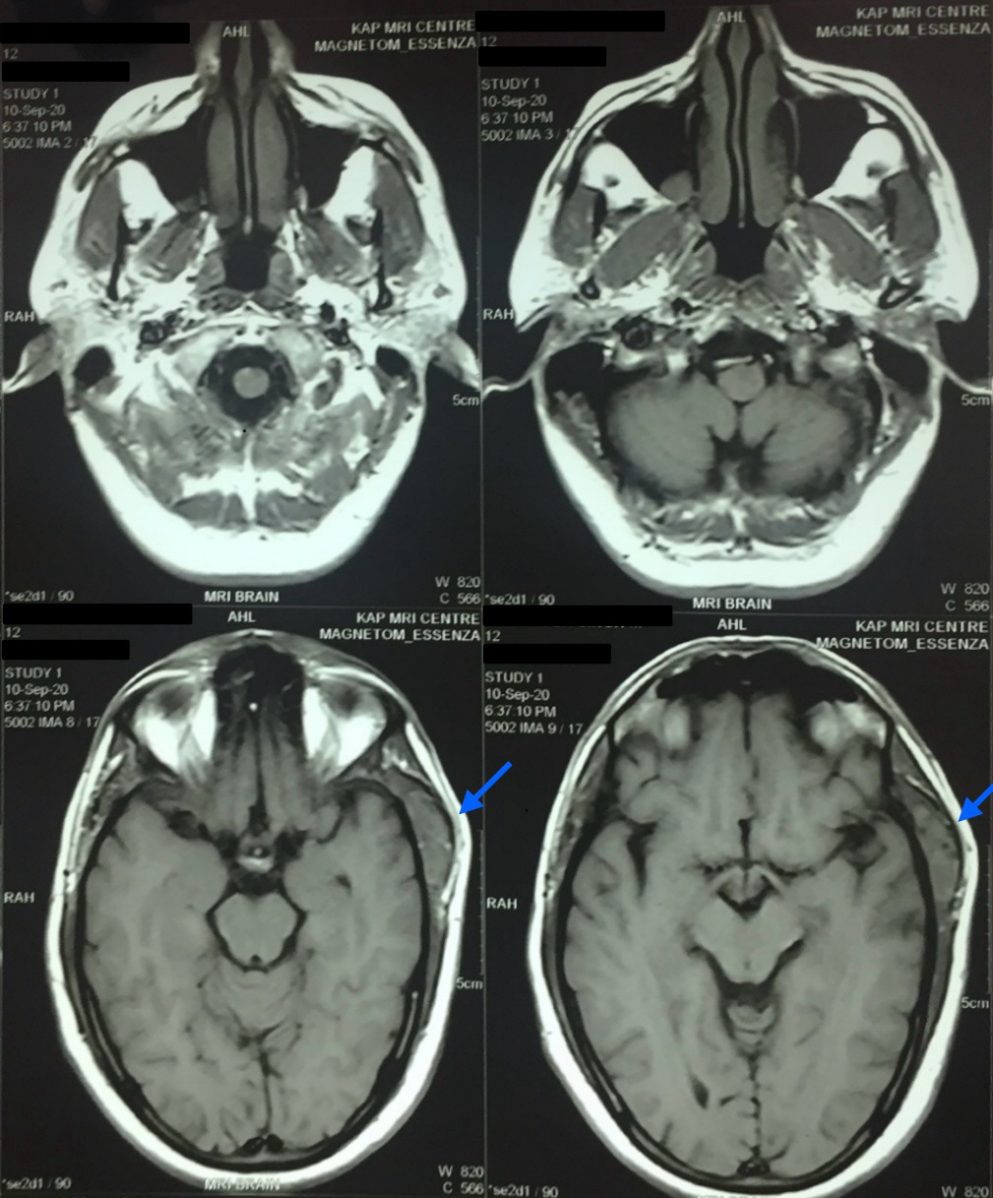
A 4.3 x 3.3 x 1.3 cm heterogenous lesion in the temporalis muscle, hyperintense on T2 weighted brain and neck MRI (axial view, indicated with blue arrows).

**Figure 5:**
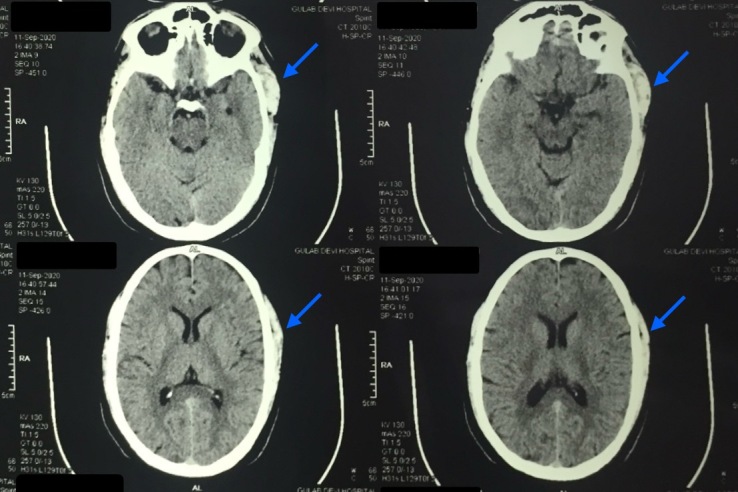
A 4.5 x 3 x 1.5 cm lobular hyperdense region with a few calcified foci in the left temporalis muscle seen on CECT (indicated with blue arrows).

On examination, the patient’s swelling was localized to the left side of the head, in the temporal region, measuring 7 cm x 5.5 cm. The patient was unable to open the mouth at the time. The swelling was soft-to-firm in consistency, non-pulsatile, and immobile and had a smooth surface without any indentation. There was localized pain and tenderness without any rise in temperature of the skin. No discoloration or facial bruises were observed. The patient’s neurological examination was otherwise insignificant. Basic laboratory investigations were unremarkable. Magnetic resonance imaging (MRI) of the head and brain revealed a well-defined 4.3 x 3.3 x 1.3 cm lesion of the temporalis region that was iso-intense on T1 and heterogeneously hyperintense on T2. The subcutaneous tissue showed a patchy area of restricted diffusion on DWI (Diffusion- Weighted Imaging). Linear areas of blooming were noted on SWI (Susceptibility-Weighted Imaging) with foci of calcification (possible phlebolith), as shown in Figures 2, 3, and 4. The lesion was further investigated with a contrast enhanced computed tomography (CECT), shown in Figure 5, that revealed a 4.5 x 3 x 1.5 cm lobular hyperdense region with a few calcified foci in the left temporalis muscle and adjoining scalp region, without extension into the underlying skull vault, heterogeneously enhancing post-contrast. No brain lesions were identified. A probable diagnosis of a vascular malformation was established.

**Figure 6:**
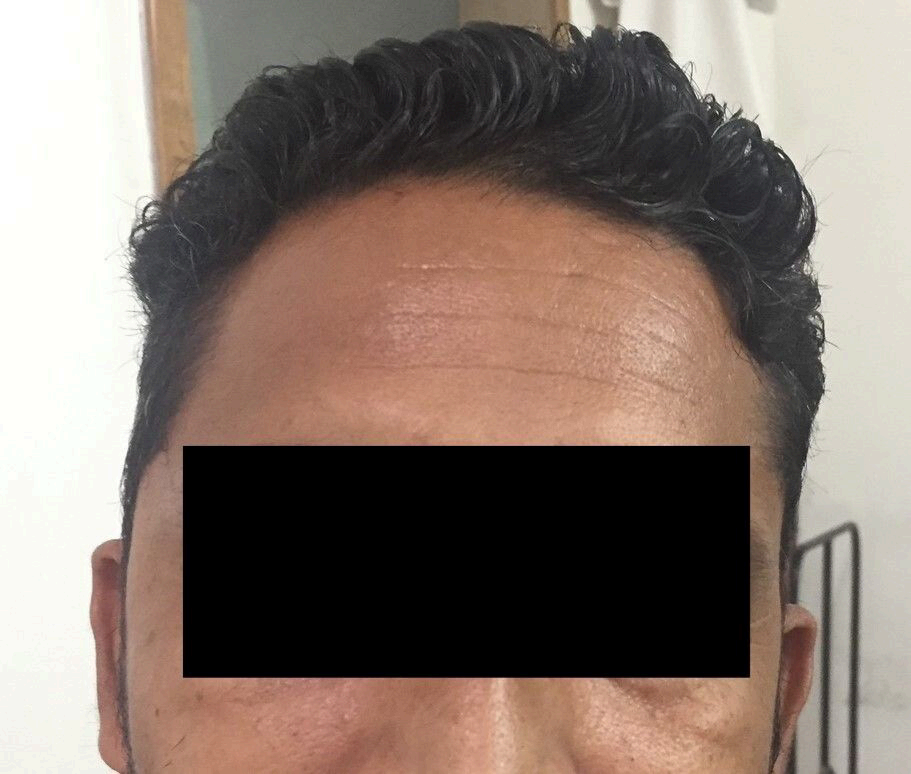
The temporal region of the patient post-self-resolution of the swelling after emotional stabilization.

The patient was advised to undergo further imaging (color flow Doppler US, angiography, Gadolinium-enhanced MRI) to plan surgical management with regards to resection or embolization. The patient refused further imaging or surgical intervention and instead opted for pharmacotherapy due to financial constraints. The patient was referred to a psychiatrist who diagnosed generalized anxiety disorder and prescribed the patient antihypertensives along with selective serotonin reuptake inhibitors (SSRIs). The patient was also recommended cognitive behavioral therapy. On follow-up, it was noted that the patient's swelling resolved spontaneously one emotional stress lessened every time and the episodes of swelling decreased in frequency following anti-anxiolytic therapy, as shown in Figure 6. The patient was advised to reconsider surgical intervention for complete resolution, but the patient was lost to follow-up.

## DISCUSSION

Our case of a patient in their 30s with a vascular malformation located in the left temporalis muscle had the unique presentation of intermittent appearance of a localized swelling during periods of intense psychological stress and anxiety in life, showing spontaneous resolution. We believe that this anomaly is one of its kind as there is no similar presentation that has been reported in the available literature.

The soft-to-firm swelling of the patient’s left temporal region was investigated with MRI and CECT. The MRI showed a well-defined lesion, iso-intense to the surrounding muscle on T1 and hyperintense on T2, helping to arrive at the probable diagnosis of vascular malformation. This is similar to how vascular malformations are described as appearing within a muscle in existing literature.^10,11^ On CECT, it appeared as a heterogeneously enhancing mass, comparable to a case presented by Kim et al.^12^ Our patient had had repeated incidences of physical trauma at the same location, leading us to believe that trauma might be the cause for development of a VM, consistent with the findings of Brown and Ward-Booth.^3^

As per the International Society for the Study of Vascular Anomalies (ISSVA), vascular malformations are broadly classified as venous, capillary, lymphatic, arteriovenous, or combined.^13^ Once diagnosed, malformations are further classified on the basis of fast or slow flow seen under color Doppler ultrasound or angiography.^14^ As our patient refused any additional investigations, further classification of the vascular malformation was difficult, although presence of intralesional calcifications does hint towards a phlebolith within a venous malformation.

One unique feature of the case was an increase in size of the swelling during periods of emotional stress followed by a pattern of resolution in absence of stress. It is known that hemodynamic stresses result in increased release of vascular growth factors. This could cause gradual dilation of a vascular malformation, eventually increasing the size of the lesion.^15^ Despite scant literature on a fluctuating VM, it is known that psychological stress can cause vasodilation and hence increased circulation in skeletal muscles.^6-8^ This mechanism could very well explain the change in size of the vascular malformation in our patient’s case.

We hope that our case can add to the existing limited literature on intramuscular vascular malformations of the temporalis muscle. It also highlights the effect of emotional stress and anxiety on vascular anomalies, encouraging more comprehensive interdisciplinary management of such cases.

## Data Availability

All data supporting the results are available as part of the article and no additional source data are required.
